# Hereditary Diseases

**Published:** 1871-03

**Authors:** 


### HEREDITARY DISEASES.

Sir Wm. Jennier thinks that where both parents are tubercular, the diathesis is intensified in their offspring. The same he believes to be the case generally in the hereditary transmission of disease. The father, he thinks, transmits a morbid tendency with more certainty than the mother does.—*Lancet.*

